# Evaluating the impact of Matrilin-1 gene polymorphisms on mandibular prognathism: A meta-analysis

**DOI:** 10.1016/j.jobcr.2025.03.019

**Published:** 2025-04-18

**Authors:** Pooja Kurmi, Prasad Nalabothu, Shubhasmita Sahoo, Henu Kumar Verma, Srinivas Gosla Reddy, L.V.K.S. Bhaskar

**Affiliations:** a Department of Zoology, Guru Ghasidas Vishwavidyalaya, Bilaspur, India; b Department of Paediatric Oral Health and Orthodontics, University Center for Dental Medicine Basel UZB, Basel, Switzerland; c Department of Immunopathology, Institute of Lungs Biology and Disease, Comprehensive Pneumology Center, Helmholtz Zentrum, Neuherberg, 85764, Munich, Germany; d GSR Institute of Cranio-Maxillofacial and Facial Plastic Surgery, Hyderabad, Telangana, India

**Keywords:** Mandibular prognathism, Matrilin, MATN1, rs20566, rs1065755, Meta-analysis

## Abstract

**Introduction:**

Matrilin-1 was shown to regulate the formation of cartilage matrix and to promote chondrocyte differentiation. This meta-analysis aims to synthesize evidence regarding the link between mandibular prognathism (MP) risk and the polymorphisms in the MATN1 gene.

**Materials and methods:**

Relevant publications were retrieved by searching the PubMed, Web of Science, and Google Scholar databases. The association between MP and the MATN1 gene polymorphisms (rs20566, rs1065755**)** was assessed by calculating odds ratios (ORs) and 95 % CIs. Between studies, heterogeneity was identified using the Cochrane Q test and I^2^ statistics. To assess the robustness of the meta-analysis sensitivity analysis was performed. The web tool MetaGenyo was used to conduct a meta-analysis.

**Results:**

A total of four Asian and one Caucasian study were eventually taken for meta-analysis. Overall, the MATN1 rs20566 and rs1065755 polymorphisms are not associated with elevated risk of MP (rs20566 AA + AG versus GG OR = 1.35, 95 % CI = 0.32–5.67; rs1065755 TT + CT versus CC OR = 2.02, 95 % CI = 0.87–4.68). The degree of heterogeneity is found to be large for the MATN1 polymorphisms (for rs20566, I^2=^89 %, and for rs1065755, I^2=^60 %).

**Conclusions:**

In conclusion, this meta-analysis did not provide evidence for the link between MATN1 polymorphisms and MP. However, the results conflict with the biological plausibility that matrilin-1 levels modulate cartilage development. Therefore, careful interpretation is needed, and further research is recommended.

## Graphical abstract

**Figure:**
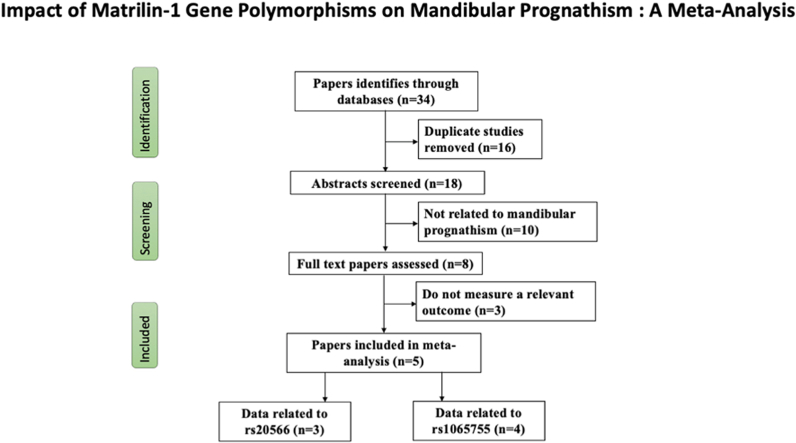

## 1 Introduction

Mandibular prognathism (MP), defined as a deformity of mandible resulting from its disproportionate growth relative to the maxilla, poses considerable challenges for individuals affected by the condition.^1^^,^^2^ Mandibular prognathism is complex inherited condition affecting both function and aesthetics. Although, severity varies from mild to extreme. The Functional Implications include masticatory dysfunction, speech difficulties, temporomandibular joint disorders and breathing issues. Further, protrusion of the mandible leads to Facial Asymmetry due to which patients may experience social anxiety. Prevalence rates of MP vary significantly across different populations ranging from 15 % in Asian population to approximately 1 % in Caucasian population.^3^ This variation suggests a potential genetic predisposition, alongside environmental influences in the development of MP, including those that regulate jaw growth and development.^4^^,^^5^ Despite the established roles of genetic and environmental factors in regulating jaw growth and development, the genetic underpinnings of MP remain poorly understood.

Recent studies have highlighted the role of extracellular matrix proteins such as matrilins, in cartilage development and maintenance as well as their potential contribution to craniofacial abnormalities.^6^ Among those, Matrilin-1known as *MATN1* located on chromosome 1p35.2. *MATN1* gene is considered as one of the most relevant candidate genes in MP. The human Matrilin-1 gene spans over 12 kilobase (kb) and contains 8 exons, 7 introns, and a 3′ untranslated region (UTR).^7^ Matrilin-1 plays an important role in the mandibular growth by regulating cartilage extracellular matrix organization and endochondral ossification.^8^ To ensure the structural integrity, MATN1 interacts with collagens and proteoglycans and thereby stabilizes the cartilage matrix.^9^ Further, Matrilin-1 plays a protective role in cartilage degeneration via TGF-β, BMPs, and SOX9, facilitating matrix deposition.^10^ Furthermore, MATN1 also determines the pace of differentiation of chondrocytes by regulating the expression and localization of Indian hedgehog (Ihh) and parathyroid hormone-related protein (PTHrP).^11^ Understanding MATN1 gene polymorphisms may help in predicting susceptibility and design targeted therapies to mandibular prognathism. Preliminary evidence has shown that the Matrilin-1 gene's single-nucleotide polymorphisms are known to contribute to the genetic susceptibility of MP in Koreans.^12^ However, the limited number of studies and occasional contradictions in the findings underscore the need for further research to elucidate these associations.

The objective of this study is to investigate the association between two specific *MATN1* polymorphisms (rs20566 and rs1065755) and the susceptibility to MP. By exploring these genetic variants, we aim to address critical gaps in the literature and provide insights into the genetic basis of mandibular prognathism. This study is significant not only in advancing the understanding the genetic cause of MP but also paving wave for future clinical research that allows for treatment of this deformity.

## 2 Materials and methods

### 2.1 Search strategy

**Fig. 1:**
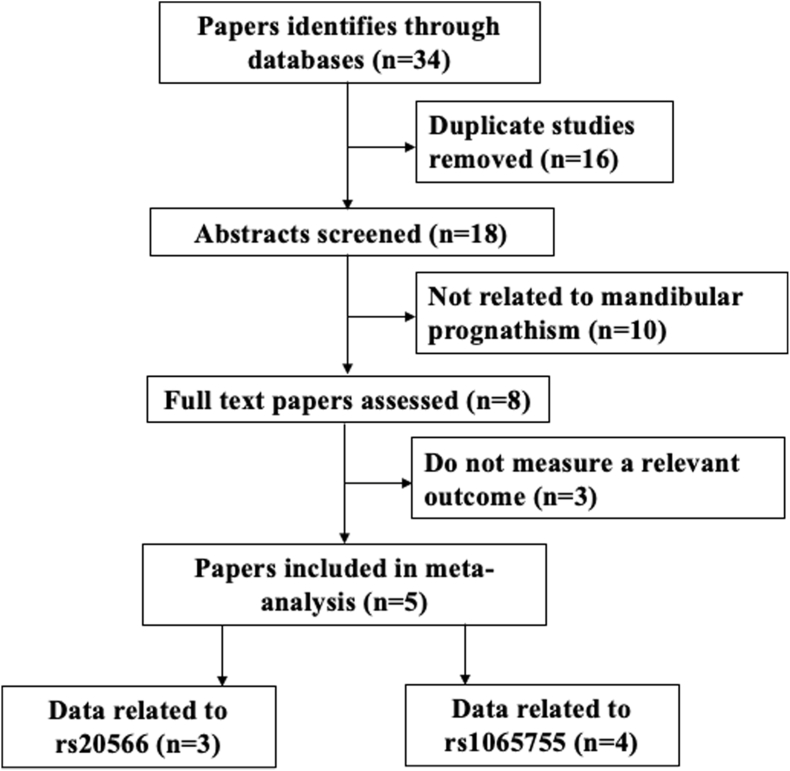
Flow diagram showing the detailed process of the literature survey following PRISMA guidelines.

Studies investigating the association between *MATN1* polymorphisms (rs20566 and rs1065755) and risk of MP were retrieved from PubMed, Web of Science and Google scholar databases. The following keywords used included “*MATN1*”, “Matrilin”1, “rs20566”, “rs1065755”, “Mandibular Prognathism” and “malocclusion”. All retrieved studies were hand-searched and selected. The final literature search was conducted on December 24, 2024. The search process followed PRISMA guidelines (Fig. 1) to ensure systematic and thorough data collection. Extracted information included author name, publication year, study location, sample population ethnicity, number of genotyped MP and control subjects, and MATN1 genotypes in both groups. Studies were included if they investigated the association between MATN1 polymorphisms and MP, provided sufficient genotype data, and were published in peer-reviewed journals. Exclusion criteria encompassed studies that lacked sufficient genotype distribution data, focused on unrelated conditions or genes or were non-peer-reviewed publications, conference abstracts, or case reports.

### 2.2 Selection of articles

The selection of eligible studies was conducted through a systematic process, which involved the removal of duplicates, followed by a screening of titles and abstracts to exclude irrelevant studies. The subsequent assessment of full texts of potentially eligible articles was based on the established inclusion and exclusion criteria.

### 2.3 Quality assessment

The quality of the included studies was evaluated using Hardy-Weinberg equilibrium (HWE) testing for the control group genotypes. In addition, factors such as study design, sample size, and population characteristics were reviewed to ensure reliability and validity.

### 2.4 Statistical analysis

The assessment of between-study heterogeneity was conducted utilizing the Cochrane's Q test and I^2^ statistics. Genotypic effect sizes were then pooled using a random-effects model to account for study heterogeneity. Finally, the results were visualized through high-resolution forest plots. A further analysis was performed using leave-one-out meta-analysis to assess the robustness of the findings.

### 2.5 Meta-analysis software

The web tool MetaGenyo: Meta-Analysis of Genetic Association Studies was used to conduct meta-analysis.^13^

## 3 Results

A total of 34 citations were retrieved from various databases, of which 16 duplicate records were removed. Abstracts of 18 studies were screened, and an additional 10 studies were excluded as they are not studying the link between MATN1 and mandibular prognathism. Subsequently, full texts of 8 papers were screened, of which 3 were excluded due to insufficient data necessary for the analysis. Finally, five studies investigating the association of *MATN1* polymorphisms (rs20566 and rs1065755) with MP were included in the meta-analysis^12^^,^^14^, ^15^, ^16^, ^17^(Fig. 1). Study characteristics were summarized in Table 1. For *MATN1* rs20566 polymorphism, 3 studies with a combined total of 246 MP patients and 204 controls met the inclusion criteria. For *MATN1* rs1065755 polymorphism, four studies involving 252 MP patients and 188 controls were analyzed. Genotype distributions in the control populations adhered to HWE proportions.

**Table 1:** Baseline characteristics of the studies included in the meta-analysis.

Author, Year	Country	Ethnicity	Genotyping method	MATN1 Genotypes					
				Mandibular Prognathism			Control		
**MATN1 rs20566**				**GG**	**AG**	**AA**	**GG**	**AG**	**AA**
Jang et al., 2010	Korea	Asian	Sequencing	68	91	5	51	57	24
Kulkarni et al., 2021	India	Asian	PCR-RFLP	14	21	0	26	4	0
Laviana et al., 2021	Indonesia	Asian	Sequencing	23	22	2	11	25	6
**MATN1 rs1065755**				**CC**	**CT**	**TT**	**CC**	**CT**	**TT**
Jang et al., 2010	Korea	Asian	Sequencing	97	55	10	90	41	1
Kulkarni et al., 2021	India	Asian	PCR-RFLP	7	28	0	19	11	0
Doke et al., 2024	India	Asian	PCR-RFLP	20	8	2	8	2	0
Toparcean et al., 2024	Romania	Caucasian	Sequencing	10	13	2	6	10	0

**Fig. 2:**
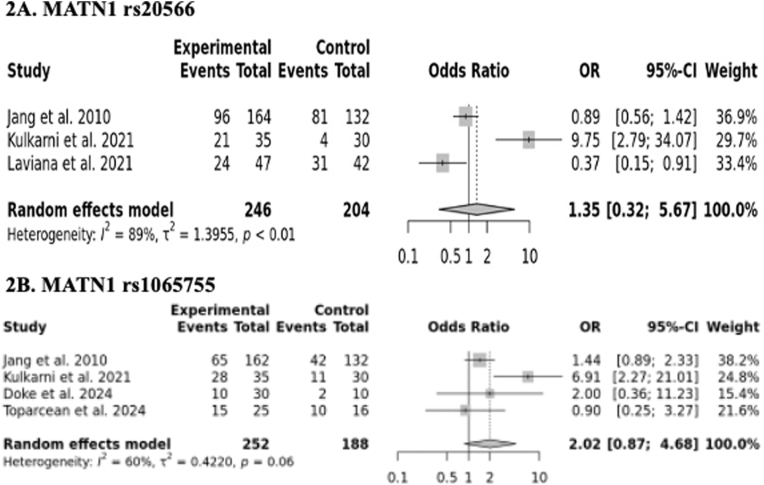
Forest plot showing pooled analysis.

**Fig. 3:**
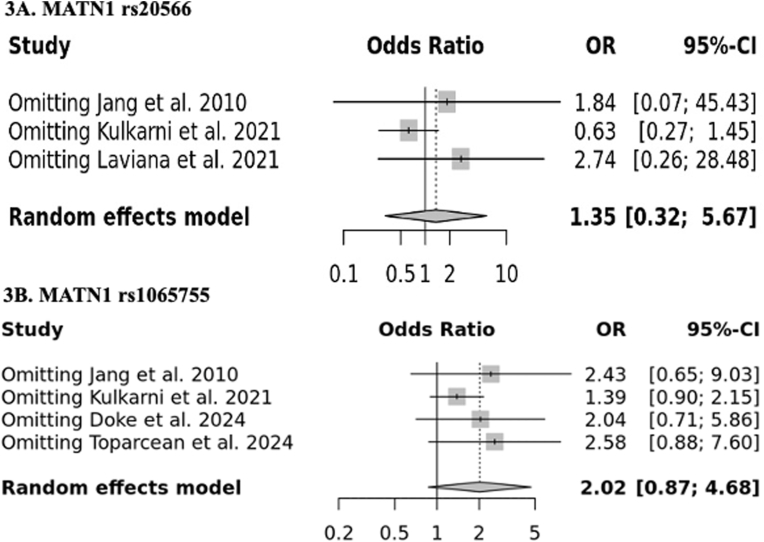
Forest plot showing sensitivity analysis.

Substantial heterogeneity was observed among studies and it falls between 60 and 100 % (for rs20566, I^2=^89 % and for rs1065755, I^2=^60 %). Due to the presence of high heterogeneity, random effect model was used to calculate the pooled OR and 95 % CI. The outcomes of the pooled analyses did not reveal a significantly elevated MP risk for rs20566 polymorphism (OR = 1.35, 95 % CI = 0.32–5.67) (Fig. 2A) and rs1065755 polymorphisms (OR = 2.02, 95 % CI = 0.87–4.68) (Fig. 2B). Further, sensitivity analysis indicated that the individual studies could not alter the pooled ORs for both *MATN1* gene polymorphisms, demonstrating that our results are statistically robust (Fig. 3A and B).

## 4 Discussion

The advent of genome technologies has revealed thousands of polymorphisms linked to health and disease. Due to their role in disease susceptibility, treatment response, and personalized dentistry, the utility of gene polymorphisms is steadily increasing in oral health. Variations in genes like 5HTR2A, DRD2, DRD3, ANKK1, COMT, MMP9, ACTN3, and ANKK1 are linked with the susceptibility to Bruxism.^18^ The COMT, HTR2A, MMPs, IL-1β, IL-6, TNF-α and ESR1 are known to influence the pain perception, inflammation, and joint integrity in temporomandibular joint disorders.^19^ Polymorphisms in TNFSF11, TNFRSF11B, WNT3A, SFRP2, LRP6, P2RX7, and LRP1 are linked with extreme post orthodontic external apical root resorption.^20^ In implant dentistry, VDR, COL1A1, RUNX2, TNF-α, OPG and RANKL gene polymorphisms are being used in personalized implant treatment.^21^

This meta-analysis synthesized evidence on the association between *MATN1* polymorphisms (rs20566 and rs1065755) and prognathism in mandible, concluding that these genetic variants are not significantly associated with an increased risk of MP. Significant heterogeneity among studies was detected. Results of sensitive analysis revealed that pooled OR is unaffected quantitatively when omitting each individual study.

Matrilin-1 is found primarily in cartilage and co-localizes with matrilin-3. Together, they are able to form heterooligomers and play a pivotal role in cartilage matrix formation and chondrocyte differentiation, both integral to craniofacial and mandibular growth.^22^,^23^ A mutation in the *MATN1* gene may alter chemical and physical properties of protein, and it is responsible for altered phenotype by itself or in combination with environmental factors. In *Equus asinus, MATN1* 503G > A genetic variation has been identified as an important genetic marker for MP.^24^ According to Rodrigues et al., *MATN1* 503G > A mutation may regulate protein synthesis, by altering splicing, elongation and maturation of RNA.^24^ In neonatal mice, Matrilin-1 was found in cranial bones, in the cartilage area of long bones and nasal septum.^25^ Studies using Matrilin-1 knockout models have shown that the matrilin modulate collagen fibrillogenesis in cartilage.^26^,^27^ Immunohistochemical study of matrilin-1 demonstrated that the matrilin-1 is highly expressed in the human condylar cartilage, particularly in arthritic disorders.^28^

A genome-wide linkage analysis in Japanese and Korean families found that the region of chromosome 1p36 where *MATN1* gene is harbored, as suggestive linkage to mandibular prognathism.^29^ *MATN1* gene polymorphisms have also been associated with idiopathic scoliosis Chinese and Korean populations.^30^, ^31^, ^32^ Subsequent association studies on MATN1 gene polymorphisms were inconclusive.^12^^,^^14^, ^15^, ^16^, ^17^ A case control study from Indian population demonstrated that the *MATN1* rs1149048 genotypes are association with mandibular retrognathism.^33^

Several lines of evidence indicated that the MATN1 gene as a key regulator of mandibular growth. Studies in donkeys linking MATN1 gene polymorphisms to mandibular prognathism susceptibility. Although, orthognathic surgery remains the standard treatment, non-surgical growth modulation may serve as a promising alternative. Hence directions focus on stem cell-based temporomandibular joint regeneration, molecular interventions, and preventive strategies through precision medicine may be considered. As gene polymorphism often varies among ethnicities, polymorphisms of the MATN1 gene and their correlation with mandibular prognathism should be delineated in other races. This would enable the utilization of MATN1 polymorphism in early diagnosis and to leverage its insights for future treatment for Class III mandibular prognathism. Compared to the other genetic disorders, mandibular prognathism is considered as aesthetic and functional issue and genetic studies were not conducted extensively. As mandibular prognathism exhibits variable expressivity and low heritability estimates with complex genetic-environmental interactions, genome-wide association studies (GWAS) using large, and well-characterized populations is warranted.

## 5 Conclusion

In conclusion, this meta-analysis did not find evidence supporting a significant association between *MATN1* gene polymorphisms and mandibular prognathism. The results should be interpreted with caution due to the limited number of studies and significant heterogeneity. The biological plausibility of matrilin-1role in cartilage development and craniofacial morphology remains compelling, necessitating further research. This study underscored the importance of integrating genetic, functional, environmental, and clinical data to unravel the complex determinants of mandibular prognathism.

## Parents/guardians consent

Parents' and guardians’ consent is not applicable as the study involves no humans.

## Sources of funding

No funding was received.

## Declaration of competing interest

The authors declare that they have no known competing financial interests or personal relationships that could have appeared to influence the work reported in this paper.
